# (1*R*,4*R*,6*S*,7*S*)-5,5-Di­chloro-1,4,8,8-tetra­methyl­tri­cyclo­[5.4.1^1,7^.0^4,6^]dodecan-12-one

**DOI:** 10.1107/S1600536813028791

**Published:** 2013-10-26

**Authors:** Ahmed Benharref, Jamal EL Karroumi, Jean-Claude Daran, Moha Berraho

**Affiliations:** a Laboratoire de Chimie Biomoléculaires, Substances Naturelles et Réactivité, URAC16, Faculté des Sciences, Semlalia, BP 2390 Bd My Abdellah, 40000 Marrakech, Morocco; b Laboratoire de Chimie de Coordination, 205 Route de Narbone, 31077 Toulouse Cedex 04, France

## Abstract

The title compound, C_16_H_24_Cl_2_O, was synthesized in three steps from β-himachalene (3,5,5,9-tetra­methyl-2,4a,5,6,7,8-hexa­hydro-1*H*-benzo­cyclo­heptene), which was isolated from essential oil of the Atlas cedar (*cedrus atlantica*). The asymmetric unit contains two independent mol­ecules with similar conformations. Each mol­ecule is built up from two fused seven-membered rings and an additional three-membered ring arising from the reaction of himachalene with di­chloro­carbene. The dihedral angles between the mean planes of the two seven-membered rings are 75.03 (9) and 75.02 (9)° in the two independent mol­ecules.

## Related literature

For the reactivity of this sesquiterpene, see: El Jamili *et al.* (2002 ▶); Lassaba *et al.* (1997 ▶). For its biological activity, see: Elhaib *et al.* (2011 ▶). For a related structure, see: Benharref *et al.* (2013 ▶). For conformational analysis, see: Cremer & Pople (1975 ▶).
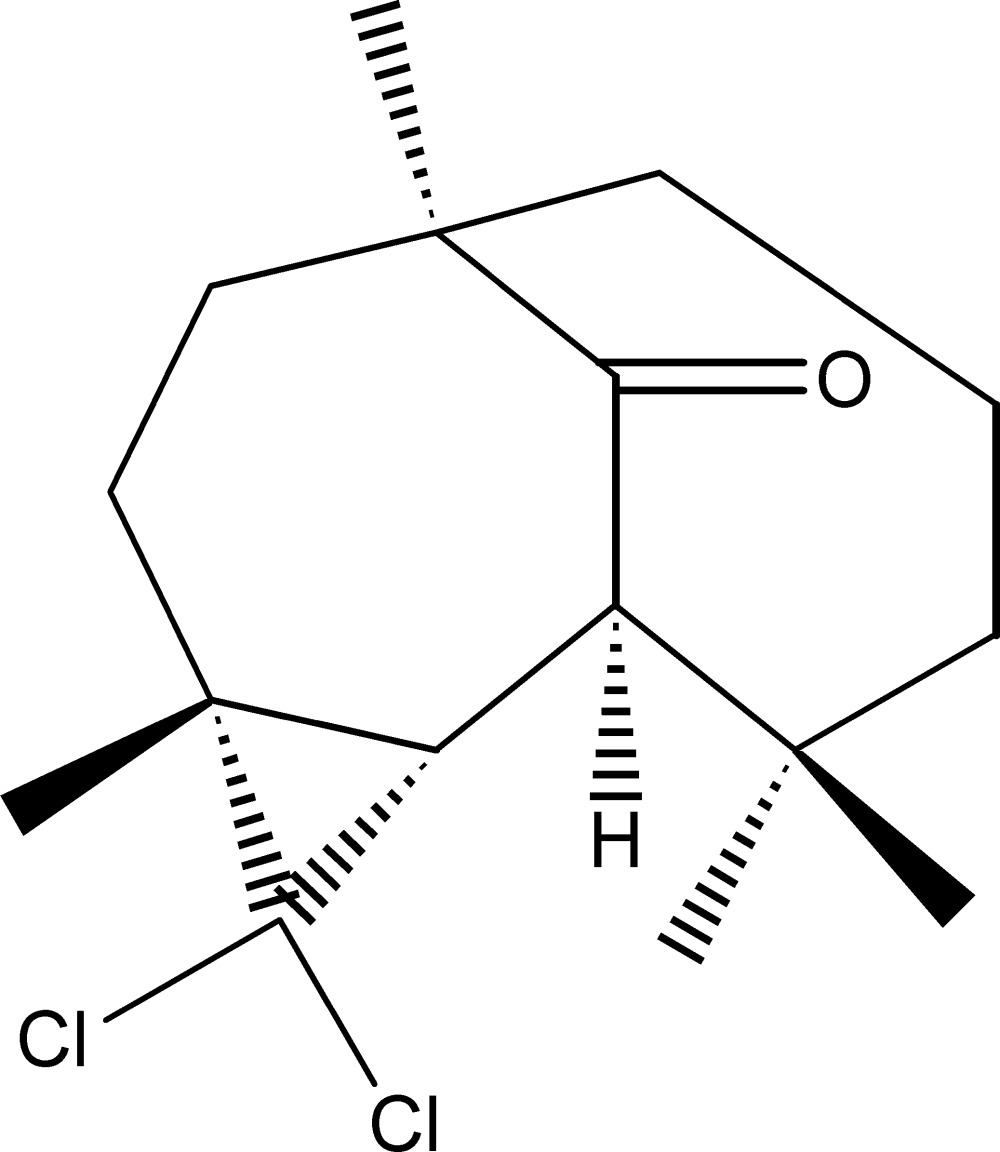


## Experimental

### 

#### Crystal data

C_16_H_24_Cl_2_O
*M* *_r_* = 303.25
Triclinic, 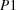

*a* = 6.5835 (2) Å
*b* = 9.2584 (3) Å
*c* = 12.8428 (5) Å
α = 85.140 (3)°
β = 84.795 (3)°
γ = 89.067 (3)°
*V* = 776.74 (5) Å^3^
*Z* = 2
Mo *K*α radiation
μ = 0.41 mm^−1^
*T* = 180 K
0.50 × 0.03 × 0.03 mm

#### Data collection

Agilent Xcalibur (Eos, Gemini ultra) diffractometer
Absorption correction: multi-scan (*CrysAlis PRO*; Agilent, 2013 ▶) *T*_min_ = 0.822, *T*_max_ = 0.988
15959 measured reflections
6301 independent reflections
5899 reflections with *I* > 2σ(*I*)
*R*_int_ = 0.030

#### Refinement

*R*[*F* ^2^ > 2σ(*F* ^2^)] = 0.030
*wR*(*F* ^2^) = 0.070
*S* = 1.05
6301 reflections
351 parameters
3 restraints
H-atom parameters constrained
Δρ_max_ = 0.19 e Å^−3^
Δρ_min_ = −0.15 e Å^−3^
Absolute structure: Flack & Bernardinelli (2000 ▶), 3127 Friedel pairs
Absolute structure parameter: 0.05 (3)

### 

Data collection: *CrysAlis PRO* (Agilent, 2013 ▶); cell refinement: *CrysAlis PRO*; data reduction: *CrysAlis PRO*; program(s) used to solve structure: *SHELXS97* (Sheldrick, 2008 ▶); program(s) used to refine structure: *SHELXL97* (Sheldrick, 2008 ▶); molecular graphics: *ORTEP-3 for Windows* (Farrugia, 2012 ▶); software used to prepare material for publication: *WinGX* (Farrugia, 2012 ▶).

## Supplementary Material

Crystal structure: contains datablock(s) I, global. DOI: 10.1107/S1600536813028791/bt6937sup1.cif

Structure factors: contains datablock(s) I. DOI: 10.1107/S1600536813028791/bt6937Isup2.hkl

Additional supplementary materials:  crystallographic information; 3D view; checkCIF report

## Figures and Tables

**Fig. 1.**
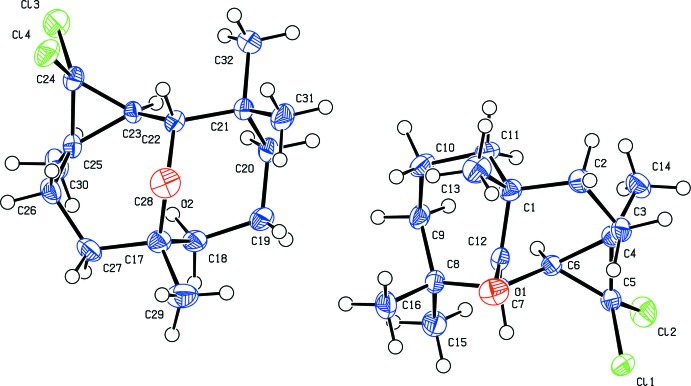
: Molecular structure of the title compound with the atom-labelling scheme. Displacement ellipsoids are drawn at the 30% probability. level. H atoms are represented as small spheres of arbitrary radii.

## supplementary crystallographic information

### 1. Comment

Our work lies within the framework of the valorization of the most abundant essential oils in Morocco, such as Cedrus atlantica. This oil is made up mainly (75%) of bicyclic sesquiterpenes hydrocarbons, among which is found the compound β-himachalene (Elhaib *et al.*, 2011). The reactivity of this sesquiterpene has been studied extensively by our team, in order to prepare new products having olfactive proprieties suitable for the perfume or cosmetics industry (El Jamili *et al.*, 2002; Benharref *et al.*, 2013). In this paper we present the crystal structure of the title compound, (1*R*,4*R*, 6S, 7S)-5,5-dichloro-1,4,8,8- tetramethyl-tricyclo[5.4.1^1^,^7^.0^4^,^6^]dodecan-12-one. The asymmetric unit of the title compound contains two independent molecules of similar geometry (Fig. 1). Each molecule contains two fused seven-membered rings, which are fused to a three-membered ring as shown in Fig. 1. In both molecules, one of the seven-membered ring has a chair conformation as indicated by the total puckering amplitude QT = 0.8377 (3) Å and spherical polar angle θ2 = 38.51 (13)° with φ2 = -100.60 (20)°, φ3 = 93.49 (18), whereas the other seven-membered ring displays screw boat conformation with QT = 1.0334 (20) Å, θ2 = 75.83 (10)°, φ2 = 151.23 (10)° and φ3 =119.77 (5)° (Cremer & Pople, 1975). Owing to the presence of Cl atoms, the absolute configuration could be fully confirmed, by refining the Flack parameter (Flack & Bernardinelli, 2000) as C1(*R*), C4(*R*), C6(S) and C7(S).

### 2. Experimental

To obtain the title compound, BF_3_—Et_2_O (1 mL) was added dropwise to a solution of (1*S*,3*R*,8*S*)-2,2-dichloro- 3,7,7,10- tetramethyltricyclo [6.4.0.0^1^,^3^]dodec-9-ene (Lassaba *et al.*, 1997) (1 g, 3.3 mmol) in 60 ml of dichloromethane at 195 K under nitrogen. The reaction mixture was stirred for two hours at a constant temperature of 195 K and the left at ambient temperature for 24 h. Water (60 ml) was added in order to separate the two phases, and the organic phase was dried and concentrated. The residue obtained was chromatographed on silica-gel eluting with hexane-ethyle acetate (97/3), which allowed the isolation of pure(1*R*,4*R*, 6S, 7S)-1,4,8,8- tetramethyltricyclo[5.4.1^1^,^7^.0^4^,^6^]dodecan-12-one in a yield of 77% (755 mg, 2.5 mmol). The title compound was recrystallized from its hexane solution.

### 3. Refinement

All H atoms were fixed geometrically and treated as riding with C—H = 0.96 Å (methyl), 0.97 Å (methylene), 0.98 Å (methine) with *U*_iso_(H) = 1.2U_eq_ (methylene, methine) or *U*_iso_(H) = 1.5*U*_eq_ (methyl).

### Crystal data

**Table:**

C_16_H_24_Cl_2_O	*Z* = 2
*M**_r_* = 303.25	*F*(000) = 324
Triclinic, *P*1	*D*_x_ = 1.297 Mg m^−^^3^
Hall symbol: P 1	Mo *K*α radiation, λ = 0.71073 Å
*a* = 6.5835 (2) Å	Cell parameters from 6301 reflections
*b* = 9.2584 (3) Å	θ = 3.1–26.4°
*c* = 12.8428 (5) Å	µ = 0.41 mm^−^^1^
α = 85.140 (3)°	*T* = 180 K
β = 84.795 (3)°	Needle, colourless
γ = 89.067 (3)°	0.50 × 0.03 × 0.03 mm
*V* = 776.74 (5) Å^3^	

### Data collection

**Table:**

Agilent Xcalibur (Eos, Gemini ultra) diffractometer	6301 independent reflections
Radiation source: Enhance (Mo) X-ray Source	5899 reflections with *I* > 2σ(*I*)
Graphite monochromator	*R*_int_ = 0.030
Detector resolution: 16.1978 pixels mm^-1^	θ_max_ = 26.4°, θ_min_ = 3.1°
ω scans	*h* = −8→8
Absorption correction: multi-scan (*CrysAlis PRO*; Agilent, 2013)	*k* = −11→11
*T*_min_ = 0.822, *T*_max_ = 0.988	*l* = −16→16
15959 measured reflections	

### Refinement

**Table:**

Refinement on *F*^2^	Secondary atom site location: difference Fourier map
Least-squares matrix: full	Hydrogen site location: inferred from neighbouring sites
*R*[*F*^2^ > 2σ(*F*^2^)] = 0.030	H-atom parameters constrained
*wR*(*F*^2^) = 0.070	*w* = 1/[σ^2^(*F*_o_^2^) + (0.0384*P*)^2^ + 0.0135*P*] where *P* = (*F*_o_^2^ + 2*F*_c_^2^)/3
*S* = 1.05	(Δ/σ)_max_ < 0.001
6301 reflections	Δρ_max_ = 0.19 e Å^−^^3^
351 parameters	Δρ_min_ = −0.15 e Å^−^^3^
3 restraints	Absolute structure: Flack & Bernardinelli (2000), 3127 Friedel pairs
Primary atom site location: structure-invariant direct methods	Absolute structure parameter: 0.05 (3)

### Special details

**Table:**

Experimental. Empirical absorption correction using spherical harmonics, implemented in SCALE3 ABSPACK scaling algorithm. CrysAlisPro (Agilent Technologies, 2013 )
Geometry. All s.u.'s (except the s.u. in the dihedral angle between two l.s. planes) are estimated using the full covariance matrix. The cell s.u.'s are taken into account individually in the estimation of s.u.'s in distances, angles and torsion angles; correlations between s.u.'s in cell parameters are only used when they are defined by crystal symmetry. An approximate (isotropic) treatment of cell s.u.'s is used for estimating s.u.'s involving l.s. planes.
Refinement. Refinement of *F*^2^ against ALL reflections. The weighted *R*-factor *wR* and goodness of fit *S* are based on *F*^2^, conventional *R*-factors *R* are based on *F*, with *F* set to zero for negative *F*^2^. The threshold expression of *F*^2^ > 2σ(*F*^2^) is used only for calculating *R*-factors(gt) *etc*. and is not relevant to the choice of reflections for refinement. *R*-factors based on *F*^2^ are statistically about twice as large as those based on *F*, and *R*- factors based on ALL data will be even larger.

### Fractional atomic coordinates and isotropic or equivalent isotropic displacement parameters (Å^2^)

**Table:**

	*x*	*y*	*z*	*U*_iso_*/*U*_eq_	
Cl2	−0.55173 (7)	0.67043 (6)	0.24182 (4)	0.03779 (14)	
Cl1	−0.12276 (7)	0.65390 (5)	0.27094 (4)	0.03107 (12)	
O1	0.1568 (2)	1.00986 (16)	0.33958 (12)	0.0348 (3)	
C7	−0.1944 (3)	0.95465 (19)	0.37125 (14)	0.0200 (4)	
H7	−0.1351	0.8613	0.4003	0.024*	
C6	−0.3450 (3)	0.9136 (2)	0.29500 (14)	0.0203 (4)	
H6	−0.4889	0.9435	0.3151	0.024*	
C5	−0.3277 (3)	0.7716 (2)	0.24622 (14)	0.0228 (4)	
C4	−0.2947 (3)	0.9096 (2)	0.17593 (14)	0.0222 (4)	
C3	−0.0806 (3)	0.9470 (2)	0.12878 (15)	0.0249 (4)	
H3A	−0.0648	0.9194	0.0557	0.030*	
H3B	0.0180	0.8884	0.1687	0.030*	
C2	−0.0269 (3)	1.1079 (2)	0.12843 (15)	0.0276 (4)	
H2A	−0.1161	1.1651	0.0821	0.033*	
H2B	0.1154	1.1214	0.0974	0.033*	
C1	−0.0476 (3)	1.1710 (2)	0.23755 (16)	0.0257 (4)	
C11	−0.2564 (3)	1.2460 (2)	0.25562 (15)	0.0257 (4)	
H11A	−0.2569	1.3356	0.2078	0.031*	
H11B	−0.3620	1.1814	0.2354	0.031*	
C10	−0.3185 (3)	1.2863 (2)	0.36775 (16)	0.0307 (4)	
H10A	−0.1950	1.3134	0.3999	0.037*	
H10B	−0.4103	1.3720	0.3646	0.037*	
C9	−0.4261 (3)	1.1633 (2)	0.43734 (15)	0.0268 (4)	
H9A	−0.5422	1.1321	0.4011	0.032*	
H9B	−0.4837	1.2034	0.5029	0.032*	
C8	−0.3000 (3)	1.0280 (2)	0.46780 (14)	0.0241 (4)	
C12	−0.0146 (3)	1.0435 (2)	0.31871 (14)	0.0229 (4)	
C14	−0.4603 (3)	0.9581 (2)	0.10671 (16)	0.0324 (5)	
H14A	−0.4543	0.8990	0.0467	0.049*	
H14B	−0.4405	1.0602	0.0815	0.049*	
H14C	−0.5936	0.9465	0.1469	0.049*	
C13	0.1222 (3)	1.2827 (2)	0.24143 (18)	0.0357 (5)	
H13A	0.1108	1.3212	0.3105	0.054*	
H13B	0.1080	1.3621	0.1870	0.054*	
H13C	0.2556	1.2356	0.2292	0.054*	
C16	−0.1382 (3)	1.0656 (2)	0.53919 (17)	0.0349 (5)	
H16A	−0.2048	1.1053	0.6021	0.052*	
H16B	−0.0451	1.1378	0.5015	0.052*	
H16C	−0.0612	0.9780	0.5597	0.052*	
C15	−0.4447 (3)	0.9173 (2)	0.52973 (16)	0.0332 (5)	
H15A	−0.5086	0.9592	0.5923	0.050*	
H15B	−0.3675	0.8299	0.5509	0.050*	
H15C	−0.5504	0.8919	0.4856	0.050*	
Cl3	−0.18678 (7)	0.88753 (6)	0.83571 (4)	0.03989 (14)	
Cl4	0.25097 (7)	0.90785 (5)	0.82503 (4)	0.03505 (13)	
O2	0.5852 (2)	0.54459 (17)	0.75362 (12)	0.0367 (4)	
C22	0.2470 (3)	0.6078 (2)	0.71903 (14)	0.0216 (4)	
H22	0.3155	0.7015	0.6938	0.026*	
C23	0.0593 (3)	0.6475 (2)	0.79047 (13)	0.0203 (4)	
H23	−0.0722	0.6174	0.7658	0.024*	
C24	0.0441 (3)	0.7881 (2)	0.84020 (15)	0.0255 (4)	
C25	0.0528 (3)	0.6489 (2)	0.91026 (14)	0.0235 (4)	
C26	0.2462 (3)	0.6118 (2)	0.96257 (16)	0.0292 (5)	
H26A	0.2271	0.6376	1.0361	0.035*	
H26B	0.3590	0.6713	0.9262	0.035*	
C27	0.3070 (3)	0.4512 (2)	0.96203 (15)	0.0305 (5)	
H27A	0.1995	0.3934	1.0048	0.037*	
H27B	0.4343	0.4364	0.9972	0.037*	
C17	0.3412 (3)	0.3893 (2)	0.85254 (16)	0.0281 (4)	
C18	0.1452 (3)	0.3153 (2)	0.82720 (15)	0.0273 (4)	
H18A	0.1271	0.2242	0.8732	0.033*	
H18B	0.0276	0.3793	0.8450	0.033*	
C19	0.1398 (3)	0.2793 (2)	0.71244 (16)	0.0316 (5)	
H19A	0.2795	0.2545	0.6839	0.038*	
H19B	0.0535	0.1931	0.7109	0.038*	
C20	0.0575 (3)	0.4033 (2)	0.64249 (15)	0.0283 (4)	
H20A	−0.0769	0.4324	0.6756	0.034*	
H20B	0.0337	0.3654	0.5749	0.034*	
C21	0.1874 (3)	0.54054 (19)	0.61824 (14)	0.0241 (4)	
C28	0.4060 (3)	0.5151 (2)	0.77276 (15)	0.0250 (4)	
C30	−0.1408 (3)	0.5985 (2)	0.97438 (16)	0.0342 (5)	
H30A	−0.1600	0.6509	1.0378	0.051*	
H30B	−0.1307	0.4942	0.9943	0.051*	
H30C	−0.2572	0.6176	0.9327	0.051*	
C29	0.5159 (3)	0.2754 (3)	0.8552 (2)	0.0421 (6)	
H29A	0.5316	0.2304	0.7885	0.063*	
H29B	0.4834	0.2009	0.9128	0.063*	
H29C	0.6434	0.3230	0.8658	0.063*	
C32	0.0631 (3)	0.6546 (2)	0.55706 (16)	0.0352 (5)	
H32A	0.0281	0.6166	0.4921	0.053*	
H32B	0.1440	0.7427	0.5400	0.053*	
H32C	−0.0622	0.6773	0.6000	0.053*	
C31	0.3815 (3)	0.5080 (2)	0.54861 (17)	0.0367 (5)	
H31A	0.4638	0.4351	0.5860	0.055*	
H31B	0.4604	0.5971	0.5317	0.055*	
H31C	0.3444	0.4711	0.4837	0.055*	

### Atomic displacement parameters (Å^2^)

**Table:**

	*U*^11^	*U*^22^	*U*^33^	*U*^12^	*U*^13^	*U*^23^
Cl2	0.0375 (3)	0.0381 (3)	0.0399 (3)	−0.0163 (2)	−0.0103 (2)	−0.0053 (2)
Cl1	0.0392 (3)	0.0217 (3)	0.0338 (3)	0.00646 (19)	−0.0096 (2)	−0.0054 (2)
O1	0.0234 (8)	0.0372 (9)	0.0440 (9)	0.0007 (6)	−0.0064 (6)	−0.0013 (7)
C7	0.0211 (9)	0.0178 (9)	0.0215 (9)	−0.0003 (7)	−0.0036 (7)	−0.0023 (7)
C6	0.0187 (9)	0.0205 (10)	0.0217 (9)	0.0014 (7)	−0.0008 (7)	−0.0024 (7)
C5	0.0226 (9)	0.0233 (10)	0.0239 (9)	−0.0027 (8)	−0.0072 (8)	−0.0043 (8)
C4	0.0231 (9)	0.0236 (10)	0.0207 (9)	0.0010 (7)	−0.0045 (7)	−0.0037 (7)
C3	0.0266 (10)	0.0281 (11)	0.0198 (9)	0.0024 (8)	0.0023 (7)	−0.0048 (8)
C2	0.0272 (10)	0.0291 (11)	0.0247 (10)	0.0011 (8)	0.0041 (8)	0.0006 (8)
C1	0.0276 (10)	0.0224 (10)	0.0261 (9)	0.0000 (7)	0.0010 (7)	−0.0008 (8)
C11	0.0278 (10)	0.0203 (10)	0.0275 (10)	0.0032 (7)	0.0013 (8)	0.0024 (8)
C10	0.0367 (11)	0.0198 (10)	0.0340 (11)	0.0047 (8)	0.0058 (9)	−0.0028 (8)
C9	0.0310 (10)	0.0242 (10)	0.0245 (10)	0.0019 (8)	0.0064 (8)	−0.0068 (8)
C8	0.0293 (10)	0.0219 (9)	0.0211 (9)	−0.0032 (8)	0.0009 (7)	−0.0044 (8)
C12	0.0234 (10)	0.0230 (9)	0.0233 (9)	0.0013 (7)	−0.0018 (7)	−0.0083 (8)
C14	0.0314 (11)	0.0407 (12)	0.0257 (10)	0.0055 (9)	−0.0076 (8)	−0.0023 (9)
C13	0.0351 (11)	0.0317 (12)	0.0381 (12)	−0.0075 (9)	0.0057 (9)	0.0014 (10)
C16	0.0458 (13)	0.0327 (12)	0.0284 (11)	−0.0061 (10)	−0.0076 (9)	−0.0091 (9)
C15	0.0411 (12)	0.0299 (11)	0.0279 (10)	−0.0047 (9)	0.0017 (9)	−0.0015 (9)
Cl3	0.0376 (3)	0.0456 (3)	0.0363 (3)	0.0189 (2)	0.0002 (2)	−0.0097 (2)
Cl4	0.0410 (3)	0.0226 (3)	0.0418 (3)	−0.0059 (2)	−0.0005 (2)	−0.0072 (2)
O2	0.0224 (7)	0.0427 (9)	0.0443 (9)	−0.0031 (6)	0.0003 (6)	−0.0027 (7)
C22	0.0211 (9)	0.0196 (9)	0.0240 (9)	−0.0014 (7)	0.0003 (7)	−0.0042 (8)
C23	0.0198 (9)	0.0224 (10)	0.0195 (9)	0.0000 (7)	−0.0032 (7)	−0.0051 (7)
C24	0.0247 (10)	0.0258 (10)	0.0260 (10)	0.0028 (8)	0.0010 (8)	−0.0071 (8)
C25	0.0257 (9)	0.0249 (10)	0.0208 (9)	−0.0028 (8)	−0.0023 (7)	−0.0067 (8)
C26	0.0347 (11)	0.0326 (12)	0.0221 (10)	−0.0056 (9)	−0.0088 (8)	−0.0051 (9)
C27	0.0364 (11)	0.0334 (12)	0.0223 (10)	−0.0049 (9)	−0.0103 (8)	0.0036 (9)
C17	0.0316 (11)	0.0239 (10)	0.0289 (11)	−0.0029 (8)	−0.0048 (8)	0.0000 (8)
C18	0.0320 (10)	0.0207 (10)	0.0288 (10)	−0.0062 (8)	−0.0037 (8)	0.0025 (8)
C19	0.0410 (12)	0.0197 (10)	0.0356 (11)	−0.0063 (9)	−0.0101 (9)	−0.0022 (9)
C20	0.0364 (11)	0.0237 (10)	0.0265 (10)	−0.0046 (8)	−0.0072 (8)	−0.0068 (8)
C21	0.0296 (10)	0.0222 (10)	0.0203 (9)	0.0024 (8)	−0.0001 (7)	−0.0036 (8)
C28	0.0245 (10)	0.0264 (10)	0.0251 (9)	−0.0020 (8)	−0.0016 (7)	−0.0086 (8)
C30	0.0348 (12)	0.0443 (13)	0.0230 (10)	−0.0085 (10)	0.0036 (8)	−0.0053 (9)
C29	0.0418 (13)	0.0338 (13)	0.0515 (15)	0.0034 (10)	−0.0157 (11)	0.0037 (11)
C32	0.0481 (13)	0.0315 (12)	0.0263 (11)	0.0062 (10)	−0.0071 (9)	−0.0011 (9)
C31	0.0466 (13)	0.0309 (12)	0.0312 (11)	0.0065 (9)	0.0081 (9)	−0.0078 (9)

### Geometric parameters (Å, º)

**Table:**

Cl2—C5	1.7679 (18)	Cl3—C24	1.7669 (19)
Cl1—C5	1.7553 (19)	Cl4—C24	1.757 (2)
O1—C12	1.213 (2)	O2—C28	1.214 (2)
C7—C12	1.525 (2)	C22—C28	1.520 (3)
C7—C6	1.529 (2)	C22—C23	1.530 (2)
C7—C8	1.566 (2)	C22—C21	1.566 (2)
C7—H7	1.0000	C22—H22	1.0000
C6—C5	1.502 (3)	C23—C24	1.496 (3)
C6—C4	1.538 (2)	C23—C25	1.536 (2)
C6—H6	1.0000	C23—H23	1.0000
C5—C4	1.507 (3)	C24—C25	1.512 (3)
C4—C14	1.507 (3)	C25—C30	1.511 (3)
C4—C3	1.516 (3)	C25—C26	1.514 (3)
C3—C2	1.536 (3)	C26—C27	1.534 (3)
C3—H3A	0.9900	C26—H26A	0.9900
C3—H3B	0.9900	C26—H26B	0.9900
C2—C1	1.557 (3)	C27—C17	1.559 (3)
C2—H2A	0.9900	C27—H27A	0.9900
C2—H2B	0.9900	C27—H27B	0.9900
C1—C12	1.533 (3)	C17—C28	1.524 (3)
C1—C11	1.538 (3)	C17—C18	1.543 (3)
C1—C13	1.542 (3)	C17—C29	1.548 (3)
C11—C10	1.536 (3)	C18—C19	1.542 (3)
C11—H11A	0.9900	C18—H18A	0.9900
C11—H11B	0.9900	C18—H18B	0.9900
C10—C9	1.528 (3)	C19—C20	1.520 (3)
C10—H10A	0.9900	C19—H19A	0.9900
C10—H10B	0.9900	C19—H19B	0.9900
C9—C8	1.534 (3)	C20—C21	1.537 (3)
C9—H9A	0.9900	C20—H20A	0.9900
C9—H9B	0.9900	C20—H20B	0.9900
C8—C16	1.530 (3)	C21—C32	1.533 (3)
C8—C15	1.532 (3)	C21—C31	1.533 (3)
C14—H14A	0.9800	C30—H30A	0.9800
C14—H14B	0.9800	C30—H30B	0.9800
C14—H14C	0.9800	C30—H30C	0.9800
C13—H13A	0.9800	C29—H29A	0.9800
C13—H13B	0.9800	C29—H29B	0.9800
C13—H13C	0.9800	C29—H29C	0.9800
C16—H16A	0.9800	C32—H32A	0.9800
C16—H16B	0.9800	C32—H32B	0.9800
C16—H16C	0.9800	C32—H32C	0.9800
C15—H15A	0.9800	C31—H31A	0.9800
C15—H15B	0.9800	C31—H31B	0.9800
C15—H15C	0.9800	C31—H31C	0.9800
			
C12—C7—C6	113.69 (15)	C28—C22—C23	115.15 (15)
C12—C7—C8	111.45 (14)	C28—C22—C21	111.30 (14)
C6—C7—C8	113.02 (14)	C23—C22—C21	112.01 (14)
C12—C7—H7	106.0	C28—C22—H22	105.9
C6—C7—H7	106.0	C23—C22—H22	105.9
C8—C7—H7	106.0	C21—C22—H22	105.9
C5—C6—C7	121.40 (15)	C24—C23—C22	121.73 (16)
C5—C6—C4	59.44 (12)	C24—C23—C25	59.80 (12)
C7—C6—C4	124.80 (15)	C22—C23—C25	124.79 (15)
C5—C6—H6	113.6	C24—C23—H23	113.4
C7—C6—H6	113.6	C22—C23—H23	113.4
C4—C6—H6	113.6	C25—C23—H23	113.4
C6—C5—C4	61.49 (12)	C23—C24—C25	61.44 (12)
C6—C5—Cl1	119.70 (13)	C23—C24—Cl4	120.37 (13)
C4—C5—Cl1	120.64 (13)	C25—C24—Cl4	120.24 (14)
C6—C5—Cl2	118.54 (14)	C23—C24—Cl3	117.87 (13)
C4—C5—Cl2	119.57 (13)	C25—C24—Cl3	120.13 (14)
Cl1—C5—Cl2	109.78 (10)	Cl4—C24—Cl3	109.71 (11)
C14—C4—C5	117.78 (16)	C30—C25—C24	117.93 (17)
C14—C4—C3	114.32 (16)	C30—C25—C26	114.40 (16)
C5—C4—C3	118.95 (16)	C24—C25—C26	118.76 (16)
C14—C4—C6	117.05 (16)	C30—C25—C23	117.56 (16)
C5—C4—C6	59.06 (12)	C24—C25—C23	58.77 (12)
C3—C4—C6	118.82 (15)	C26—C25—C23	118.41 (16)
C4—C3—C2	114.24 (16)	C25—C26—C27	113.38 (16)
C4—C3—H3A	108.7	C25—C26—H26A	108.9
C2—C3—H3A	108.7	C27—C26—H26A	108.9
C4—C3—H3B	108.7	C25—C26—H26B	108.9
C2—C3—H3B	108.7	C27—C26—H26B	108.9
H3A—C3—H3B	107.6	H26A—C26—H26B	107.7
C3—C2—C1	115.69 (15)	C26—C27—C17	116.55 (16)
C3—C2—H2A	108.4	C26—C27—H27A	108.2
C1—C2—H2A	108.4	C17—C27—H27A	108.2
C3—C2—H2B	108.4	C26—C27—H27B	108.2
C1—C2—H2B	108.4	C17—C27—H27B	108.2
H2A—C2—H2B	107.4	H27A—C27—H27B	107.3
C12—C1—C11	113.36 (16)	C28—C17—C18	112.73 (15)
C12—C1—C13	108.50 (16)	C28—C17—C29	108.98 (17)
C11—C1—C13	109.14 (16)	C18—C17—C29	108.93 (17)
C12—C1—C2	106.37 (15)	C28—C17—C27	107.26 (16)
C11—C1—C2	110.02 (16)	C18—C17—C27	110.05 (16)
C13—C1—C2	109.37 (16)	C29—C17—C27	108.80 (17)
C10—C11—C1	116.28 (17)	C19—C18—C17	115.65 (17)
C10—C11—H11A	108.2	C19—C18—H18A	108.4
C1—C11—H11A	108.2	C17—C18—H18A	108.4
C10—C11—H11B	108.2	C19—C18—H18B	108.4
C1—C11—H11B	108.2	C17—C18—H18B	108.4
H11A—C11—H11B	107.4	H18A—C18—H18B	107.4
C9—C10—C11	113.05 (16)	C20—C19—C18	113.13 (17)
C9—C10—H10A	109.0	C20—C19—H19A	109.0
C11—C10—H10A	109.0	C18—C19—H19A	109.0
C9—C10—H10B	109.0	C20—C19—H19B	109.0
C11—C10—H10B	109.0	C18—C19—H19B	109.0
H10A—C10—H10B	107.8	H19A—C19—H19B	107.8
C10—C9—C8	117.89 (16)	C19—C20—C21	118.06 (16)
C10—C9—H9A	107.8	C19—C20—H20A	107.8
C8—C9—H9A	107.8	C21—C20—H20A	107.8
C10—C9—H9B	107.8	C19—C20—H20B	107.8
C8—C9—H9B	107.8	C21—C20—H20B	107.8
H9A—C9—H9B	107.2	H20A—C20—H20B	107.1
C16—C8—C15	108.00 (16)	C32—C21—C31	107.83 (16)
C16—C8—C9	110.18 (16)	C32—C21—C20	108.17 (16)
C15—C8—C9	107.78 (16)	C31—C21—C20	110.29 (16)
C16—C8—C7	109.33 (15)	C32—C21—C22	107.76 (15)
C15—C8—C7	107.83 (15)	C31—C21—C22	109.32 (15)
C9—C8—C7	113.54 (14)	C20—C21—C22	113.29 (15)
O1—C12—C7	119.24 (17)	O2—C28—C22	119.31 (17)
O1—C12—C1	119.90 (17)	O2—C28—C17	120.21 (17)
C7—C12—C1	120.83 (15)	C22—C28—C17	120.45 (15)
C4—C14—H14A	109.5	C25—C30—H30A	109.5
C4—C14—H14B	109.5	C25—C30—H30B	109.5
H14A—C14—H14B	109.5	H30A—C30—H30B	109.5
C4—C14—H14C	109.5	C25—C30—H30C	109.5
H14A—C14—H14C	109.5	H30A—C30—H30C	109.5
H14B—C14—H14C	109.5	H30B—C30—H30C	109.5
C1—C13—H13A	109.5	C17—C29—H29A	109.5
C1—C13—H13B	109.5	C17—C29—H29B	109.5
H13A—C13—H13B	109.5	H29A—C29—H29B	109.5
C1—C13—H13C	109.5	C17—C29—H29C	109.5
H13A—C13—H13C	109.5	H29A—C29—H29C	109.5
H13B—C13—H13C	109.5	H29B—C29—H29C	109.5
C8—C16—H16A	109.5	C21—C32—H32A	109.5
C8—C16—H16B	109.5	C21—C32—H32B	109.5
H16A—C16—H16B	109.5	H32A—C32—H32B	109.5
C8—C16—H16C	109.5	C21—C32—H32C	109.5
H16A—C16—H16C	109.5	H32A—C32—H32C	109.5
H16B—C16—H16C	109.5	H32B—C32—H32C	109.5
C8—C15—H15A	109.5	C21—C31—H31A	109.5
C8—C15—H15B	109.5	C21—C31—H31B	109.5
H15A—C15—H15B	109.5	H31A—C31—H31B	109.5
C8—C15—H15C	109.5	C21—C31—H31C	109.5
H15A—C15—H15C	109.5	H31A—C31—H31C	109.5
H15B—C15—H15C	109.5	H31B—C31—H31C	109.5
